# Deep-rooted Indian Middle Palaeolithic: Terminal Middle Pleistocene lithic assemblage from Retlapalle, Andhra Pradesh, India

**DOI:** 10.1371/journal.pone.0302580

**Published:** 2024-08-27

**Authors:** Devara Anil, Monika Devi, Gopesh Jha, Zakir Khan, Vrushab Mahesh, P. Ajithprasad, Naveen Chauhan

**Affiliations:** 1 Department of Archaeology and Ancient History, Maharaja Sayajirao University of Baroda, Vadodara, India; 2 Luminescence Laboratory, AMOPH Division, Physical Research Laboratory, Ahmedabad, Gujarat, India; 3 Indian Institute of Technology, Gandhinagar, Gujarat, India; 4 Department of Archaeology, Max Planck Institute of Geoanthropology, Jena, Germany; 5 Institute for Archaeological Sciences, Eberhard-Karls-Universität Tübingen, Tübingen, Germany; 6 School of Studies in Ancient Indian History, Culture, and Archaeology, Pt. Ravishankar Shukla University, Raipur, Chhattisgarh, India; New York University, UNITED STATES

## Abstract

The Indian Middle Palaeolithic has been recognized as crucial evidence for understanding the complex behavioural dynamics of hominins and is also seen as a behavioural marker of early *Homo sapiens* in the region. Recent research has pushed back the timeline of the Middle Palaeolithic to the Middle Pleistocene epoch, indicating a potential *in-situ* emergence from the earlier Late Acheulian culture. The long-lasting Middle Palaeolithic culture in India evolve over multiple glacial-interglacial cycle, showing signs of behavioural resilience to bigger climatic upheaval like ~74 ka Toba super-eruption. This has added to the complexity of our understanding of the Middle Palaeolithic in the region and emphasizes the need for further research. This study focuses upon the investigation of Middle Palaeolithic artefacts found in the Retlapalle area within the upper Gundlakamma river basin, Andhra Pradesh. The dating of the artefact-bearing layer was carried out using the p-IR-IRSL method, which revealed a burial age of 139±17 thousand years. The Retlapalle assemblage is characterized by a diverse range of Levallois core reductions, various retouched artefacts, with a dominance of pointed tools, and a few blade components. The study provides a valuable addition to the existing body of data concerning Palaeolithic sites dating back to the Middle Pleistocene, a period that remains relatively underexplored.

## Indian Middle Palaeolithic: A reappraisal

The Middle Palaeolithic has attracted much attention in the last few decades which not only altered our understanding but also added several new perspectives on the hominin behavioural evolution in the region. The Middle Palaeolithic in India was first identified in 1956 following the flake tool discoveries by H.D. Sankalia at Nevasa on the Pravara riverbank in Maharasthra [1]. In the early stages of Middle Palaeolithic research, these flake-based industries were ascribed with different names such as Middle Palaeolithic [2], Middle Stone Age [3], Series II [1], Nevasian [4] and Flake culture [5]. A large number of sites belonging to the Middle Palaeolithic culture have been identified in the region after its identification as a distinct cultural entity [6]. The chronology of Middle Palaeolithic culture in the early stages was developed based on the stratigraphic association of it with preceding Acheulian culture. The sites at Lakhampur West and East [7], the Kortallayar basin sites [8] as well as excavated sequences from Bhimbetka [9], Indola-ki-Dhani and Singi Talav [10], suggest that the Late Acheulian Industries are succeed by the Middle Palaeolithic with little evidence for occupation hiatus between the two cultural phases. The chronometric ages from 16R dune places the transition from Late Acheulian to Middle Palaeolithic in South Asia between ⁓390–150 ka [11] whereas the end of the Middle Palaeolithic was estimated to be between ⁓40–25 ka based on the evidence of early Upper Palaeolithic dated to ⁓25 ka from 16 R Dune [11] and Patne [12].

**Fig 1:**
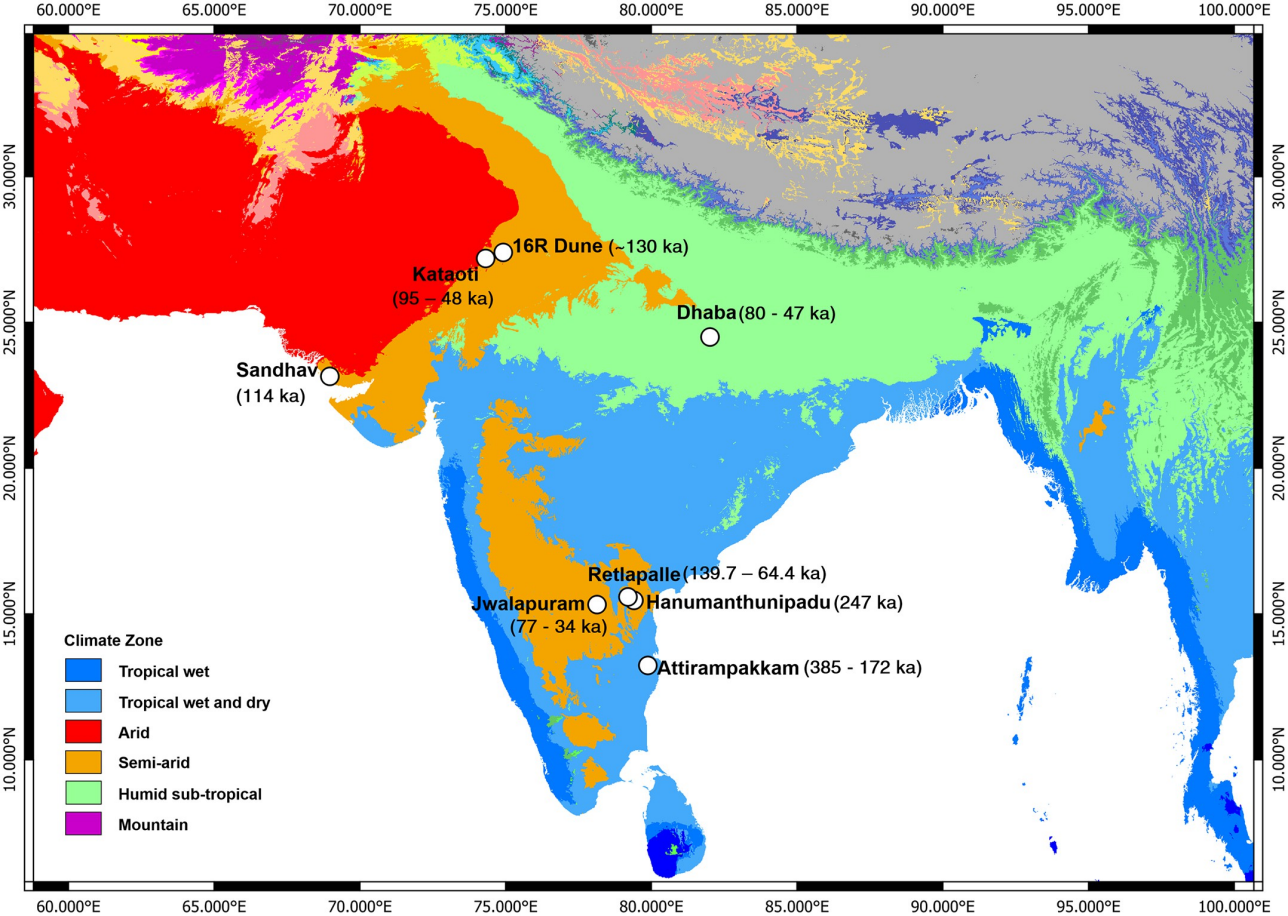
Köppen-Geiger climate map showing location of dated Middle Palaeolithic sites in India. Spatial distribution of Middle Palaeolithic sites shows varying climatic context of Levalloisian assemblages in India. (Credit: The map is visualized using QGIS, adapting open access Köppen-Geiger climate map at https://koppen.earth/).

However, from the beginning of the 21st Century (from 2005 onwards), there has been fairly good number of chronometric ages available for the Middle Palaeolithic culture in India (Fig 1). Jwalapuram in Andhra Pradesh reveals multiple Middle Palaeolithic artifact-bearing localities spanning 80 to 38 ka [13]. Notably, the association between Middle Palaeolithic artefacts and Youngest Toba Tuff (YTT) deposits was a distinctive feature of Jwalapuram. The Middle Palaeolithic assemblages from Jwalapuram are argued to be similar to the African MSA assemblages and the former were introduced to the region by the incoming *Homo sapiens* from Africa.

Further, a continuity in the Middle Palaeolithic technology from below and above YTT sediments suggests no or minimal impact of Toba eruption on hominin behaviour in India [14]. Apart from Jwalapuram, a few more sites have yielded chronometric ages for the Middle Palaeolithic sites, which include Kataoti in Rajasthan [14], Sandhav in Kachchh [15], and Dhaba in Madhya Pradesh (Fig 1) [16]. All these dates place the Indian Middle Palaeolithic in the Late Pleistocene epoch and corroborates the pre-Toba model, which argues that the Middle Palaeolithic in the region was a product of *Homo sapiens* dispersals into Eurasia [13].

**Fig 2:**
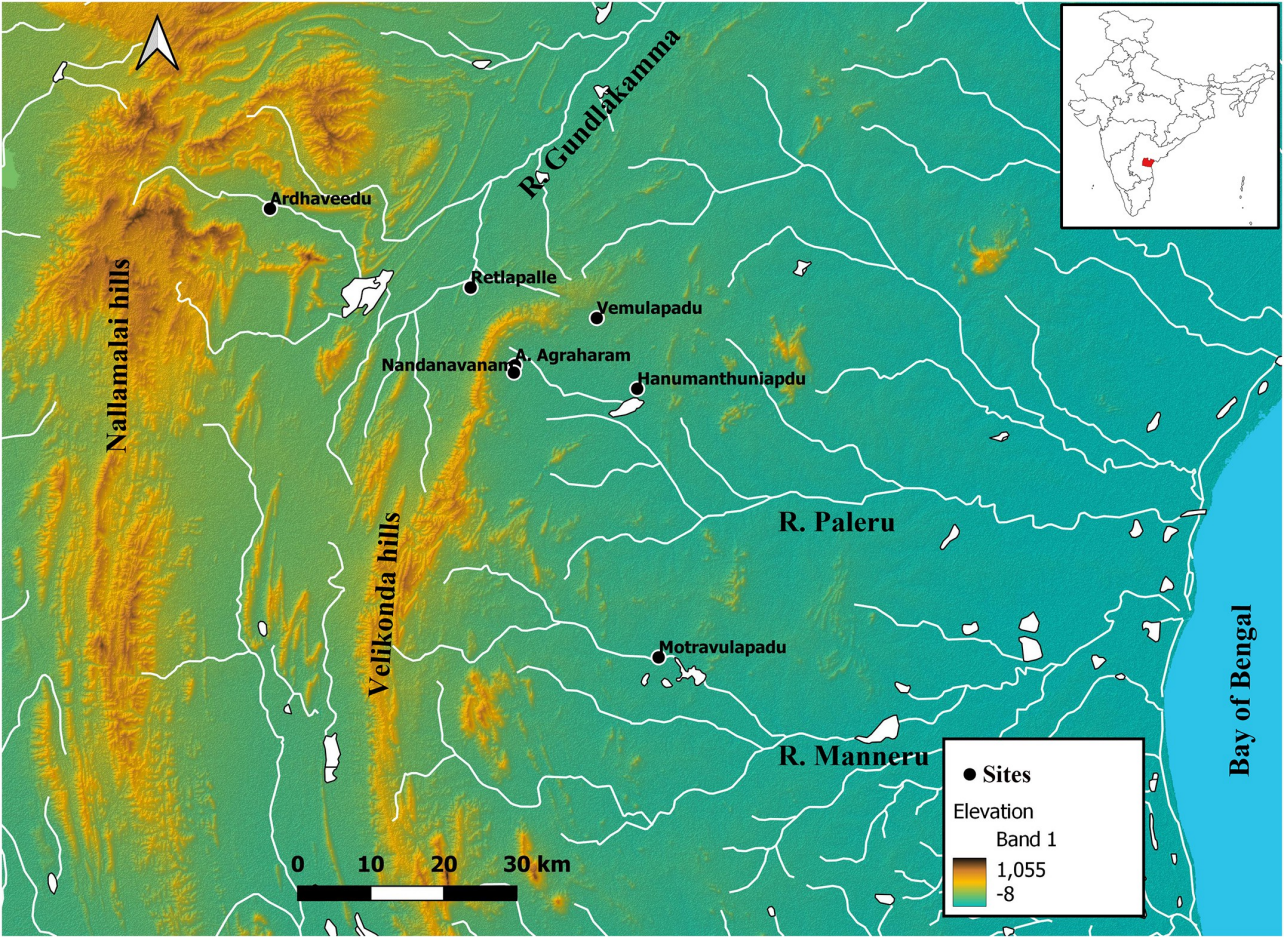
Map showing the location of Retlapalle and other sites in the Gundlakamma basin. (Credit: The map is visualized in QGIS using SRTM dataset from https://earthexplorer.usgs.gov/.

These studies have highlighted the significance of the Indian Middle Palaeolithic in the global discussions on the topic of *Homo sapiens* dispersals in Eurasia [17]. Further, the evidence for the youngest Acheulian in India, dated to the beginning of Late Pleistocene suggests the continuity of archaic hominins in the region until the appearance of *Homo sapiens* [18]. These two sets of evidence act as opposite narratives to the early ideas of Late Acheulian-Middle Palaeolithic transition in region that can be dated between 350–150 ka [11]. However, recent studies from the southern part of India provided both chronological and technological evidence for the local development of Middle Palaeolithic technology from the preceding Late Acheulian, placing it the same in the Middle Pleistocene epoch (Fig 2) [19, 20]. However, these new finds need to be supported with more chronometric ages for the Middle Palaeolithic assemblages within the Middle Pleistocene epoch.

In this context, we present the chronology and technology of the Middle Palaeolithic assemblages from Retlapalle site in the upper Gundlakamma river in Andhra Pradesh, India. The Middle Palaeolithic artefacts at this site are associated with carbonate rich silty sediments are dated to 139±17 ka using the Luminescence method. Thus, this terminal Middle Pleistocene Middle Palaeolithic assemblage adds another significant data point to Indian Palaeolithic studies. The lithic assemblage from Retlapalle is characterised by the diverse Levallois reductions, dominance of points, and a few blade components.

### Retlapalle site

Retlapalle (RTP) (15.590630°N, 79.194290° E) is located on the right bank of the stream Erravagu, a tributary of the Gundlakamma River in the Prakasam District of Andhra Pradesh, India (Fig 2). The Gundlakamma River originates in the Nallamalai ranges. It flows eastwards and forms two large lakes at Cumbum and Markapur, before altering its course between north-east and south-east and draining into the Bay of Bengal.

Recent Quaternary geological studies have identified the presence of volcanic ash concentrated in the upper and middle reaches of the river and attributed it to YTT based on geochemical studies done through the XRF spectroscopy [19]. Deposits of YTT have also been reported from adjacent river systems, including the Jurreru [13] and the Sagileru [20, 21]. Archaeological surveys of the Gundlakamma valley were first undertaken in the 1950s, indicating the presence of a broad range of Palaeolithic activity within the landscape. Lower Palaeolithic (Acheulean) and Middle Palaeolithic occurrences are reported as widespread in the Gundlakamma valley, alongside the reported presence of an Upper (Late) Palaeolithic site at Yerragondapalem [22].

**Fig 3:**
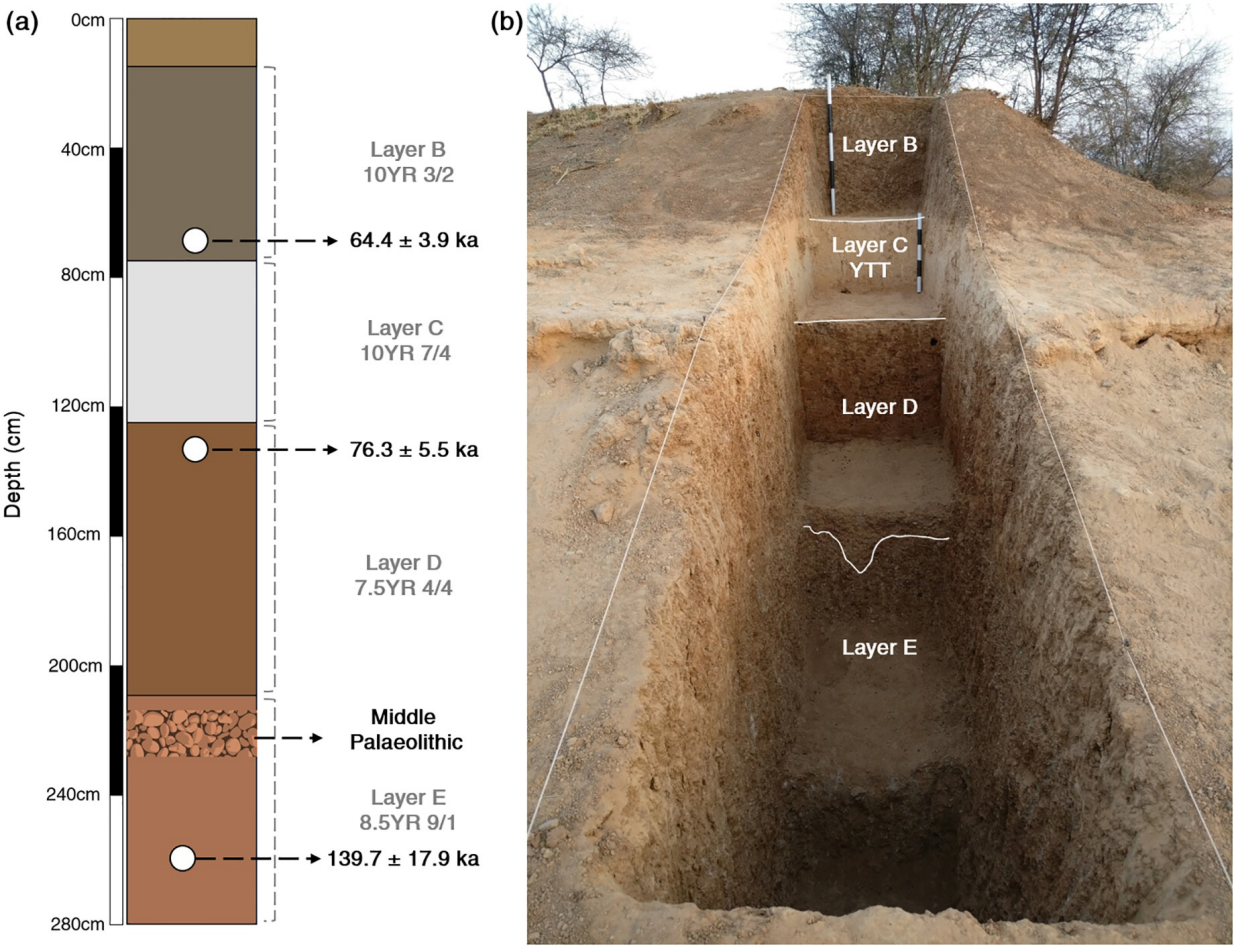
Stratigraphy of Retlapalle: (a) Composite lithostratigraphy of fluvial sequence in Upper Gundlakamma basin, showing geochronological data; (b) Photograph of Retlapalle step-trench, showing exposed litho-units at the site.

Recent surveys by the current team have identified a further six sites with volcanic ash horizons and 20 new Palaeolithic sites in the upper Gundlakamma valley, including the site of Retlapalle (N15.59°, E79.19°) on the bank of a minor tributary named the Erravagu, which includes both: an ash horizon and an artefact bearing horizon [24, 25]. Wherever the ash beds are present, they have a maximum thickness of 50 cm and appear as laterally discontinuous horizons, disrupted by post-depositional erosion [24]. Preliminary analyses of stone tool assemblages indicate the widespread presence of Middle Palaeolithic technologies that are closely comparable to the Middle Palaeolithic assemblages known from the nearby Jurreru Valley [23]. To place these archaeological results within a chronostratigraphic framework, we conducted excavations at Retlapalle, where survey results indicated the presence of ash deposits and Middle Palaeolithic artefacts (Fig 3). Based on our observations at 26 Palaeolithic sites and multiple geological sections across the upper reaches of the Gundlakamma river basin we reconstructed the composite stratigraphy (Unit A to E). These observations were supported by the stratigraphic units (Units B to E) exposed in an excavated trench at Retlapalle. Unit A was not present in the trench at Retlapalle but can be seen at other places at the site.

## Materials and methods

All necessary permits were obtained for the described study, which complied with all relevant regulations. Excavation permissions are obtained from Archaeological Survey of India. Excavations were conducted using single context recording system by dividing the sediments into 10 cm spits. Sediments were collected at 10 cm intervals for geoarchaeological analysis and from each distinct sedimentary layer for OSL age estimations. Luminescence ages was estimated using the protocols as per [25]. Analysis of the lithic artefacts employed standard terminologies frequently used across South Asia (e.g., [13, 14, 16, 24]. Sedimentological analysis of samples from the step trench was discussed in [25].

## Results

### Luminescence chronology

**Fig 4:**
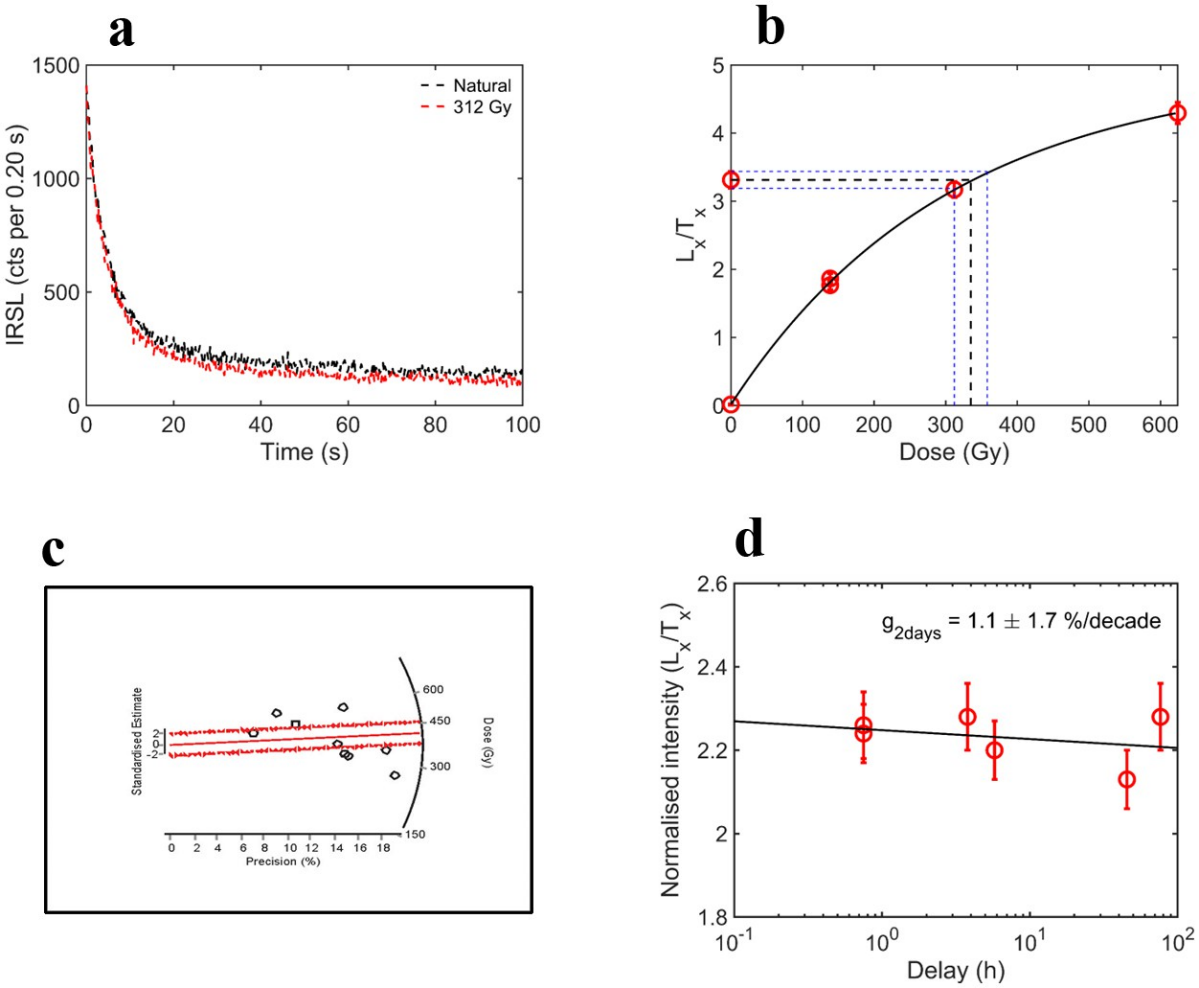
Results of p-IR-IRSL analyses of sample RTP-18-4 from Unit E. a: typical feldspar shine down curve; b: typical dose response curve; c: radial plot representing the estimated palaeodoses; d: typical g-value data.

Sediment samples from Units B, D and E from the Retlapalle step trench were dated using the post-infrared infrared-stimulated luminescence (pIR-IRSL) method. The age of Unit B, which is above YTT, is 64.4±3.9 ka, and the age for the sample from Unit D underlying the YTT is 76.3± 5.5 ka (25). The sample from Unit D shows relatively higher over-dispersion (30%) than the samples from Unit B and D (Fig 3). This may be due to the presence of rich carbonates. However, for D_e_ estimation of central age model (CAM) was used and Unit E yielded an age of 139.7±17.9 (Figs 3 and 4 and S1 Table). No significant fading was observed in the sample; therefore, the age was not fading corrected.

### Lithic assemblage

From the step-trench (within Unit E), 101 lithics were collected, most of them being debitage, flakes and flaked pieces (79%). Finished artefacts consist of one unidirectional core, one diminutive biface, and two retouched points (Table 1). These were embedded within the calcrete horizon, and a carbonate crust on the surface of the artefacts was observed. Informally retouched artefacts, consisting of retouched flakes (n = 6) and flaked pieces (n = 6), are also present in the assemblage. A 10 x 10 m grid was laid out adjacent to the step-trench to increase the artefact sample size and better understand the assemblage technology. Only one artefact horizon was identified in the step-trench, and surface observations also indicated the same. Therefore, artefacts collected from the surface grid and trench are treated as a single assemblage for analysis. Typo-technological classification of the assemblage from step trench and surface grid is presented in Table 1.

**Table 1:** Composition of the assemblage recovered from step-trench and surface grid.

Type	Trench	%	Grid	%	Total	%
**Cores**						
Recurrent Levallois core	0	0.0	4	1.7	4	1.2
Preferential Levallois core	0	0.0	6	2.6	6	1.8
Unidirectional Recurrent Levallois Core	0	0.0	1	0.4	1	0.3
Discoidal core	0	0.0	9	3.9	9	2.7
Radial Core	0	0.0	7	3.1	7	2.1
Blade core	0	0.0	4	1.7	4	1.2
Unidirectional core	1	1.0	0	0.0	1	0.3
Core fragment	5	5.0	12	5.2	17	5.2
**Total Cores**	**6**	**5.94**	**43**	**18.78**	**49**	**14.85**
	
**Retouched**						
Bifacial Point	0	0.0	11	4.8	11	3.3
Unifacially Retouched Point	0	0.0	2	0.9	2	0.6
Retouched Point	2	2.0	3	1.3	5	1.5
Tang Point	0	0.0	5	2.2	5	1.5
End scraper	0	0.0	3	1.3	3	0.9
Round scraper	0	0.0	2	0.9	2	0.6
Side Scraper	0	0.0	1	0.4	1	0.3
Notch	0	0.0	3	1.3	3	0.9
Retouched blade	0	0.0	2	0.9	2	0.6
Retouched flake-blade	0	0.0	2	0.9	2	0.6
Retouched flake	6	5.9	26	11.4	32	9.7
Retouched piece	6	5.9	10	4.4	16	4.8
Chopper	0	0.0	1	0.4	1	0.3
Diminutive biface	1	1.0	1	0.4	2	0.6
Diminutive Cleaver	0	0.0	4	1.7	4	1.2
**Total Retouched**	**15**	**14.85**	**76**	**33.19**	**91**	**27.58**
	
**Unretouched**						
Levallois flake	0	0.0	6	2.6	6	1.8
Blade	0	0.0	9	3.9	9	2.7
Flake-blade	0	0.0	3	1.3	3	0.9
Flake	30	29.7	27	11.8	57	17.3
Flaked piece	29	28.7	16	7.0	45	13.6
Debitage (< 2 cm in length)	21	20.8	49	21.4	70	21.2
**Total Unretouched**	**80**	**79.21**	**110**	**48.03**	**190**	**57.58**
**Total**	**101**	**100**	**229**	**100**	**330**	**100**

**Fig 5:**
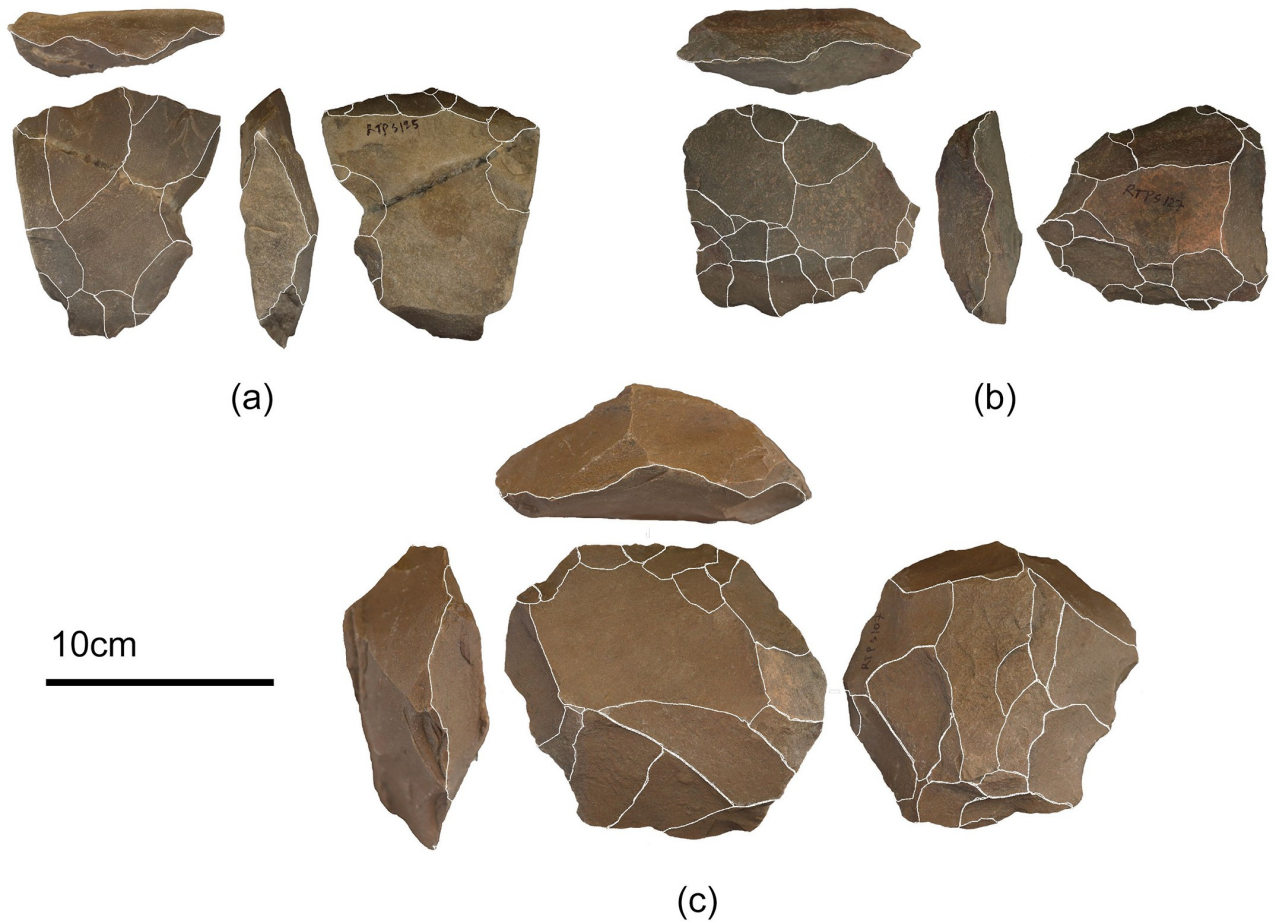
Levallois reductions from the assemblage. A: Unidirectional Recurrent Levallois Core; b: Recurrent Levallois Core; c: Preferential Levallois Core.

**Fig 6:**
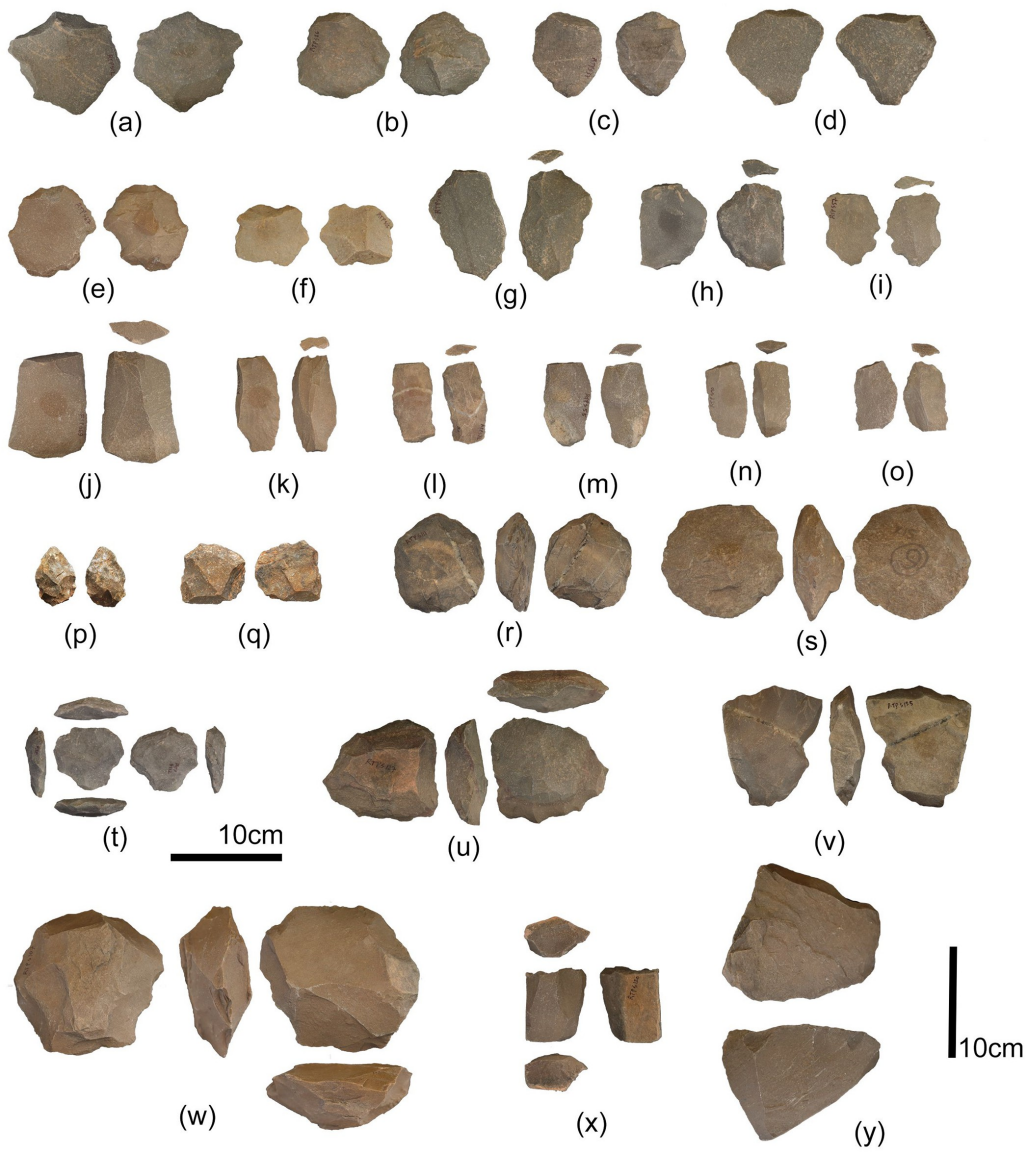
Composite lithic illustrations from Layer E assemblage. a to e: Prepared core flakes; f to i: Levallois flakes; j to o: Blades; p: Diminutive hand axe; q: Multiplatform core; r and s: Discoidal Cores; t and u: recurrent Levallois cores; V: Levallois point core; w: preferential Levallois core; x and y: blade cores.

Most artefacts do not contain cortex (81%), indicating that the initial preparation of cores must have taken place off-site near raw material sources. As the site Retlapalle is located on the bank of a tributary stream, Erravagu, pebbles from the streambed must have served as raw material sources. Except for three artefacts showing evidence of slight abrasion, the rest of the assemblage is in fresh condition. Most of the artefacts from the step trench show a thin crust of calcrete on the surface of the artefacts, whereas this was limited only to a few artefacts collected from the surface, which probably washed off due to the recent surface exposure. The number of cores in the assemblage accounts for 49, most of which belong to formal reduction strategies, including Levallois, blade, discoidal, radial, and unidirectional. Notably, except for 17 core fragments, no other informal cores are present in the assemblage. Among Levallois core reduction strategies, recurrent and preferential systems are present along with one example of Unidirectional Recurrent Levallois core (Figs 5 and 6). Nine discoidal cores are present in the assemblage, among which four cores are less than 5 cm in length. Four blade cores present in the assemblage have parallel flake scars. Seven radial cores display centripetal radial reduction.

A maximum of three and a minimum of two core rotations were observed in Levallois cores, whereas discoidal and radial cores show a maximum of four and a minimum of three rotations (Table 2). Blades and unidirectional cores show single rotation. Most cores show two (n = 12) and three (n = 11) major scars (more than 1/3 core length), with three cores exhibiting one, four cores with four, five, and six scars each. Cores’ mean length, medial width and medial thickness are 67.56x70.12x34.03 mm when oriented along the flaking axis (last flake scar). The core’s proximal, medial, and distal widths measured along the flaking axis are 62.60x70.12x52.77 mm, respectively. The proximal shape of the cores is relatively straight (mean 0.89 mm), whereas the distal shape is tapering (mean 1.39 mm).

**Table 2:** Statistical data for core attributes.

Attribute	N	Mean	SD	Min.	Max.
Length	32	67.56	16.78	41.20	114.45
Proximal Width	32	62.60	20.68	26.17	109.19
Medial Width	32	70.12	19.15	37.90	123.84
Distal Width	32	52.77	17.74	26.35	98.87
Proximal Thickness	32	29.77	26.99	7.81	141.83
Medial Thickness	32	34.03	20.22	13.41	119.87
Distal Thickness	32	25.91	13.53	10.46	77.86
Proximal Shape	32	0.89	0.15	0.67	1.29
Distal Shape	32	1.39	0.30	1.00	2.28
Elongation	32	0.98	0.20	0.63	1.47
Flatness	32	2.35	0.66	0.86	4.22
No. of Core Rotations	32	2.75	0.84	1.00	4.00
Last Platform Angle	32	72.34	8.71	50.00	95.00
No. of Major Flake Scars	32	2.72	1.11	1.00	6.00
No. of Flake Scars	32	4.84	1.37	3.00	8.00
No. of Feather terminations	32	1.41	0.91	0.00	4.00
No. of non-feather Terminations	32	1.34	1.15	0.00	4.00
Last Scar Length	32	41.46	16.12	18.17	77.58
Last Scar Width	32	31.40	13.53	10.84	81.90
Last Scar Elongation	32	1.42	0.44	0.65	2.45

No considerable variation in core elongation (ranging between 0.63–1.47) is observed, maybe because there is no significant variation in the cores’ mean length and mean medial width. The core flatness index ranges from 0.90 to 4.49, where most cores are wider than thick. Three cores have cortical platforms, 15 have single conchoidal platforms, and 14 show multiple conchoidal platforms. Platforms were facetted on 20 cores; in 12 instances, no preparation was observed. The last scar face length of cores is less than the axial core length, indicating the flaking face is limited to the smaller axial surface. The last scar lengths range from 18.17–77.58 mm, with an average of 41.46 mm, and the last scar widths range between 10.84–81.90 mm, and a mean of 31.40 mm. On average, the last flake scar elongation indicates that relatively square-shaped flakes were removed (mean = 1.42). Half of the last flake scars exhibit feather terminations (50.57%), with non-feather terminations accounting for 49.42%. One hundred forty-seven flakes, including both retouched and unretouched, have been recorded in the assemblage, including 112 complete flakes. Flakes were classified according to technological type to understand their position in the reduction sequence (Table 3). Typo-technology of the flakes was described based on complete flakes.

**Table 3:** Technological breakdown of the flakes in the assemblage.

Technological Type	Unretouched	%	Retouched	%	Total	%
Blade	7	11.86	2	3.77	9	8.04
Core preparation flake	25	42.37	18	33.96	43	38.39
Roughout flake	5	8.47	0	0.00	5	4.46
Prepared core flake	12	20.34	26	49.06	38	33.93
Levallois flake	7	11.86	5	9.43	12	10.71
Flake-blade	2	3.39	1	1.89	3	2.68
Eclat deborant	1	1.69	0	0.00	1	0.89
Kombewa flake	0	0.00	1	1.89	1	0.89
**Total**	**59**	**100**	**53**	**100**	**112**	**100**

A wide range of technological diversity is evident amongst the flakes, from core preparation flakes to end products. Few roughout flakes (n = 5) indicate that initial preparation of the cores was done off-site; however, core preparation flakes account for 38%, suggesting that later stages of core reduction were carried out on-site. The presence of one platform rejuvenation sample also corroborates the observation mentioned above. End products such as Levallois flakes, and prepared core flakes account for 55% and dominate the flake component (Fig 5). Blades and elongated flakes also form a considerable (3%) percentage in the assemblage.

The most common dorsal scar patterns present on complete flakes in the assemblage are radial (32.1%), followed by proximal (31.2%) and weakly radial (25.8%). Flakes with no dorsal scars (cortical flakes) were present in 3.5%, and on 7.1% of the flakes, it was difficult to determine the dorsal scar pattern. Cortical coverage ranges from 0–100%, with 83% of flakes recorded with no cortex and 12.5% with less than 50% cortex present. Low frequencies of flakes with only cortical platforms (5.3%) and 100% cortical cover of the dorsal and platform surface (4.4%) are observed. Most flakes present feather terminations (91%), followed by step (4.4%) and indeterminate (4.4%) terminations. Mean axial flake dimensions are 57.2 x 47.7 x 13.5 mm; on average, flakes are oval/squarish in shape (mean elongation = 1.30) (Table 4).

**Table 4:** Statistical data for the flake attributes.

Attribute	N	Mean	SD	Min.	Max.
Length	112	57.20	22.81	17.37	193.14
Proximal Width	112	42.32	17.68	12.67	108.77
Medial Width	112	47.73	17.56	11.39	113.05
Distal Width	112	38.42	18.08	6.54	120.49
Medial Thickness	112	13.59	4.22	5.25	28.32
Elongation	112	1.30	0.69	0.62	6.81
Flatness	112	3.59	1.01	1.10	6.97
Proximal Shape	112	0.89	0.19	0.43	1.42
Distal Shape	112	1.36	0.45	0.72	3.40
Platform width	112	35.89	17.36	5.67	110.27
Platform Thickness	112	12.53	5.10	3.53	41.26
Platform area	112	499.93	412.16	20.02	2581.42
Platform Angle	112	74.64	9.95	55.00	110.00
Dorsal scar count	112	2.84	1.25	0.00	6.00
No. Unidirectional arrises	112	0.41	0.67	0.00	2.00
No. Radial arrises	112	1.06	1.09	0.00	4.00

Typical flakes are more than four times wide as thick (mean flatness = 3.59), with a maximum range of 1.10–6.97. 75.8% of the flakes exhibit slightly expanding proximal margins (mean proximal shape index = 0.89), with 24.1% exhibiting contracting proximal margins, leading to an upper proximal shape index of 1.14. In contrast, the distal shape indicates that 77.6% of the flakes exhibit distal contracting margins (mean = 1.48). Single conchoidal platforms are the most common type (44.6%), followed by multiple conchoidal (41%), dihedral (6.2%), and cortical (5.3%) types. Platform preparation is dominated by facetted platforms with 47.3%, followed by overhang removal (25%), unprepared platforms (24.1%) and indeterminate (3.5%). A wide range in platform size is evident, with platform width ranging from 5.67–110.27 mm and platform thickness ranging from 3.53–41.2 mm. The platform shape index indicates that platforms are typically elongated (mean = 2.91), with 90% being two times wider than thick. A total of 45 artefacts categorized as flaked pieces are present, which bear no precise ventral morphologies or negative flake scars originating from the margins of the artefacts but have clearly undergone some reduction.

Ninety-one retouched artefacts, including Levallois points, scrapers, bifacial points and retouched pieces, are recorded in the assemblage (Fig 6). A wide range of retouched artefacts is present in the assemblage, including one chopper, two diminutive bifaces and four diminutive cleavers. Retouched pieces are also present in small quantities. Among the retouched category, informally retouched flakes, blades, flake-blades, and pieces collectively dominate with 15.8%. Points, including bifacial, retouched, and tang points, form 7% of the assemblage’s second dominant category in retouched artefacts. Scrapers and notches form 2.7% of the assemblage. Prepared core flakes were the most preferred type to make retouched artefacts forming 50% of the total retouched artefacts. Retouch length measured on the retouched artefacts shows that half of the artefacts (51.8%) are randomly retouched, whereas, on 48.2% of the artefacts, retouch was regular. The average retouch length was 63.7%, ranging between 17.6–107.6 mm. The location of the retouch was recorded and divided between distal and lateral margins, both distal and lateral margins and random. 29.6% of the artefacts were retouched on both distal and lateral margins, followed by retouching on the distal end and random locations forming similar percentages (27.7%) and lateral margins (14.8%). Retouch type was also recorded as the flake surface on which retouch was done (dorsal, ventral, or both sides). Most of the time, retouching was observed on the dorsal surface (44.4%) and both dorsal and ventral surfaces (44.4%), followed by the ventral surface (11.1%). Notably, 11 bifacial points were noted in the assemblage with length, width, and thickness dimensions as 72.32x50.90x20.83 mm, respectively.

## Discussion

### Technological insights

The Middle Palaeolithic artefacts from Retlapalle provide significant insights on the evolution of Middle Palaeolithic technology during terminal Middle Pleistocene in India. Core attribute analysis offers several insights into reduction intensity and strategy at Retlapalle. A relatively low proportion of cores with cortical surfaces, including platform surfaces, suggest that the primary reduction was conducted away from the site, possibly at the raw material procurement area. Levallois core types (Fig 5) suggest that some reduction sequences were conducted principally at the site. The fewer variation in the size of flakes indicates that preliminary reduction activities were performed off the site. In addition, most flakes present minimal cortical cover that supports the abovementioned observation. Average flake dimensions typically fall between 13–57 mm, and the largest and smallest flakes present at the site are likely to have been by-products of general reduction activity rather than an outcome of specific reduction strategies. However, the presence of Levallois flakes and small debitage (less than 2 cm in length) indicates that some specific debitage products were produced at the site (Fig 6). Platform types are typically complex, and platform preparation is commonly observed. Of all the scar patterns, proximal and radial flakes dominate, indicating that radial flaking was employed to create flakes with large perimeters. A few blade core reduction products were also present in the assemblage. Diversification in the Levallois technology was also observed in the assemblage with preferential, recurrent, and point cores. Notably, considerable number of points including bifacial, retouched and Levallois points were identified in the assemblage. Retouched artefacts were more abundant in the assemblage compared to the nearby Middle Palaeolithic assemblage at Hanumanthunipadu, dated to >247 ka [26].

### One technology, different niche

**Fig 7:**
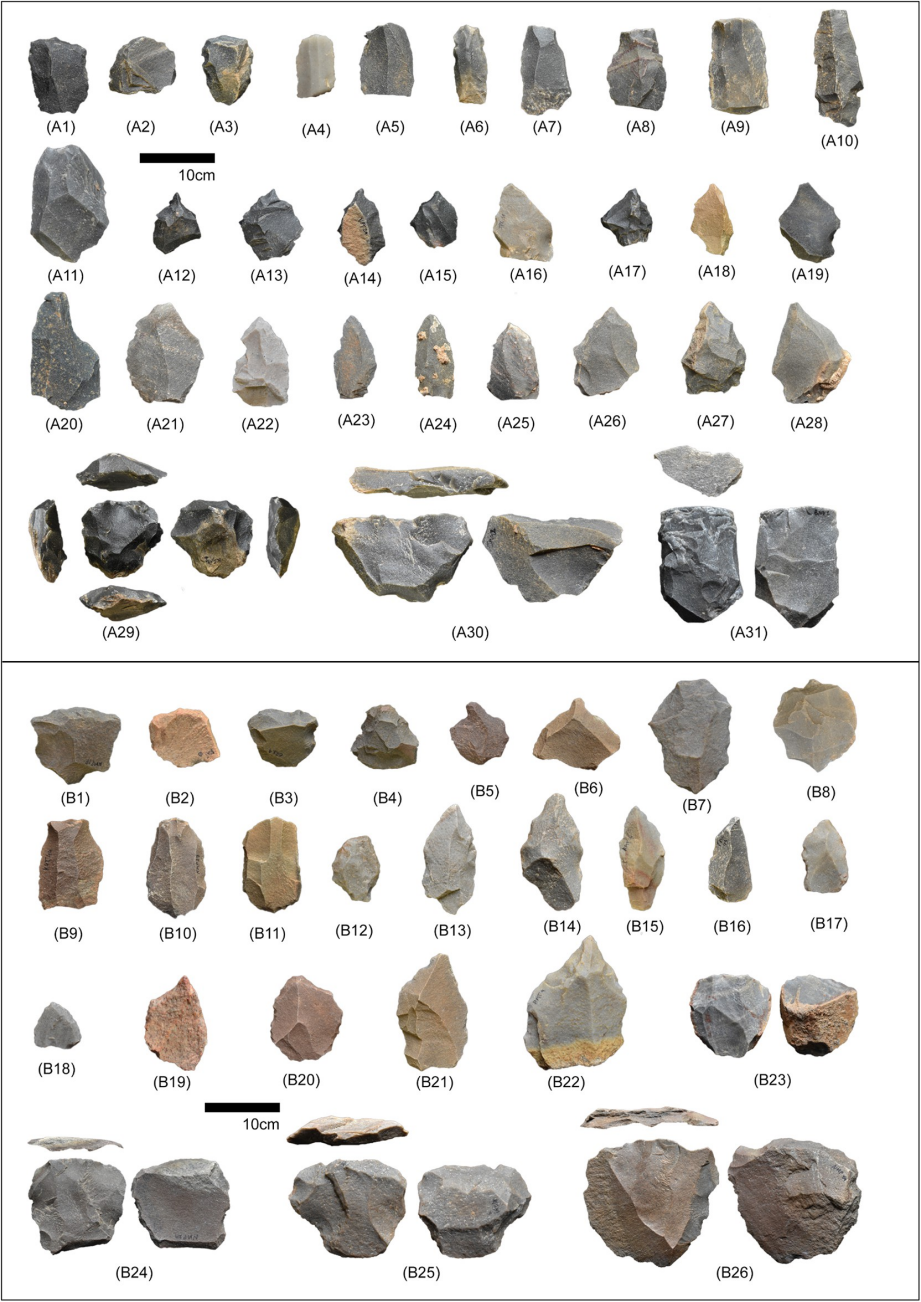
Middle Palaeolithic tools from Layer B (post-YTT; all of these artefacts are surface collections from 11 different sites associated with Layer B of composite stratigraphy of Gundlakamma river basin (see ref. 23 and 25 for more details) and shown together for representative purposes): A1—A3: scrapers, A4 –A10; blades, A11 –Levallois flake, A12 –A15: borers, A16 –A19: tanged points; A20-A28: Levallois points; A29: recurrent Levallois core, A30: unidirectional core, and A31: blade core. Middle Palaeolithic tools from Layer D (B1 –B26; pre-YTT: all of these artefacts are surface collections from 12 different sites associated with Layer D and shown together for representative purposes of composite stratigraphy of Gundlakamma river basin (see ref. 23 and 25 for more details) and shown together for representative purposes): B1 –B4: scrapers, B5 –B6: borers, B7—B8: Levallois flakes, B9 –B11: blades, B12 –B15: tanged points, B16 –B17: Levallois points, B18 –B22: retouched points, B23: bidirectional Levallois core, B24: unidirectional core, and B25 –B26: recurrent Levallois cores.

The site Retlapalle (RTP) yields Middle Palaeolithic assemblages dated to 139±17 ka and YTT deposits dated between 76–64 ka [25]. From the excavations at the site, four stratigraphic layers were delineated from Layer B to E, where Layer E is the bottom most layer. The upper three units of RTP represent Late Pleistocene deposits where layer B can be equated to MIS4 (64 ka) and layer D (76 ka) can be associated to MIS5 [25]. The YTT yielding layer C represents the terminal phase of MIS5. Sedimentological datasets indicate that the Late Pleistocene deposits of RTP is characterized by low-energy fluvial conditions, probably represent a pond or shallow depression [25]. This observation is further supported by mineral magnetic studies showing no major excursions in the sediments’ magnetic properties, indicating no palaeosol formations in the upper part of the stratigraphic sequence during Late Pleistocene. Earlier we have reported the presence of Middle Palaeolithic assemblages in layers B and layer D from the adjoining area (upper reaches of Gundlakamma river basin), which showed long and continuous Middle Palaeolithic occupation in the region (Fig 7) [25].

**Fig 8:**
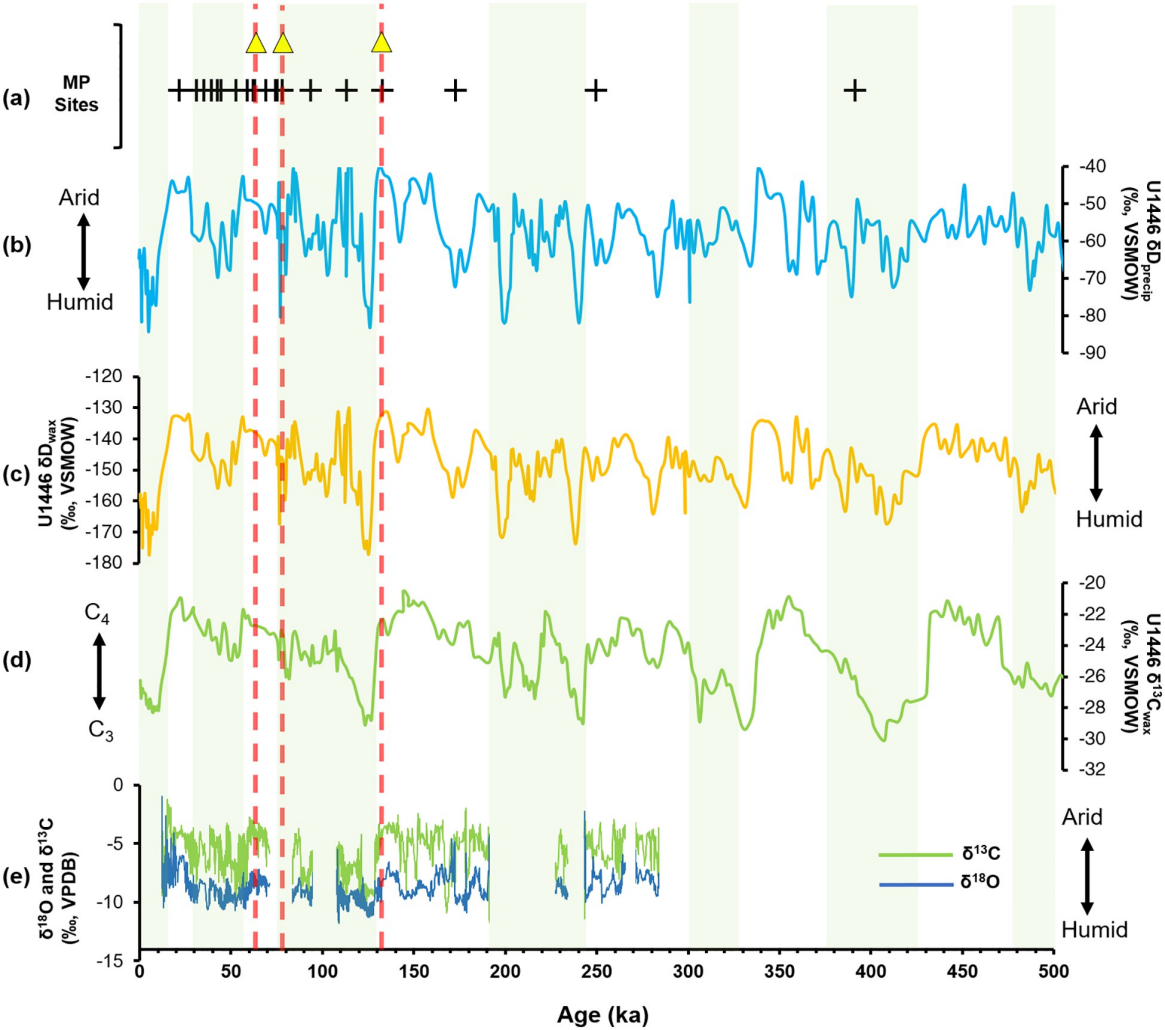
Occurrence of Indian MP sites across glacial-interglacial conditions show highly variable palaeoclimatic context of MP assemblages: (a) Dated Middle Palaeolithic sites in India; (b—d) Compound-specific δ^13^C and δD measurements of long-chain plant-wax compounds from Mahanadi fan (Bay of Bengal), demonstrating shifts in vegetation and precipitation patterns during different glacial-interglacial transitions in last 500 ka (28); (e) δ^18^O and δ^13^C record from Bitto cave speleothem in N. India also compliments Mahanadi dataset (27).

Newly dated Middle Palaeolithic assemblage from layer E, extends Middle Palaeolithic occupation in the upper Gundlakamma river basin to terminal Middle Pleistocene epoch (MIS 6). Loss On Ignition (LOI) data indicate that layer E is primarily enriched in carbonate and contains a relatively low percentage of organic content [26]. The concentration of carbonates in the layer E, relatively higher than in the later litho-units, suggest that the hominins occupied the site during arid to semi-arid conditions [26]. Similar signs of aridity were also observed in another paleoclimate record from Bitto cave (Uttarakhand) and U1448 δ^13^C_wax_ record from Mahanadi basin (Orissa) in the Indian subcontinent, corresponding to MIS 6 stage (Fig 8) [27, 28].

The study region shows major differences in paleoclimatic context, corresponding to MIS 6 and 4 (two glacial stages) and MIS5 (last interglacial phase) associated with Middle Palaeolithic occupations [25]; current study). The existing Middle Palaeolithic data also show varying environmental context of Middle Palaeolithic occupation in the Indian subcontinent, forcing us think about the complex nature of hominin-environment relationship in the region (Fig 8) [17, 29]. The overall spatial distribution of Middle Palaeolithic sites shows heterogeneity, indicating the prime role of the degree and tempos of climatic impact on local environment [29]. Such variability in the paleoenvironmental context of Middle Palaeolithic sites may indicate habitat variability and ecological plasticity of Middle Palaeolithic hominin populations in the Indian subcontinent. Future research on on-site palaeoenvironmental reconstructions will allow us to test different models of the climate-environment-human relationship at Retlapalle and surrounding regions.

### Conclusion: Retlapalle, SAMP and global narratives

The shift from large heavy-duty tools of the Acheulean techno-complex to smaller, standardized, and sophisticated Middle Palaeolithic techno-complex occurred during a very critical junction in human evolution [30–33]. It is argued that the emergence of Levallois technology occurred, primarily in response to increased environmental variability around later phases (between MIS 9–12) of Middle Pleistocene [33–36]. Potts et al. [37] demonstrate that the African MSA locally emerged from the preceding Acheulean techno-complex, coinciding with a period of high environmental variability and large-scale landscape changes. Continental Europe also presents a similar story, associating glacial conditions of MIS 8 with the asynchronous, gradual and diffused evolution of Levallois technology from pre-existing bifacial techno-complex [30]. Recent research indicates that the emergence of Middle Palaeolithic technology at different regions are associated with different hominin species, suggesting convergent evolution of Middle Palaeolithic trajectories across the old world [31]. Similar patterns of parallel emergence and evolution of Levallois technology is also observed in India [26, 32]. The last decade of Palaeolithic research in India have shown evidence of deep-rooted history of Middle Palaeolithic, lasting for more than ~350 kyrs in the Indian subcontinent. Similar intertwined and diffused relationship is observed between Acheulean and Middle Palaeolithic techno-complex, showing gradual changes at assemblage level over a long course of time [26, 32, 38]. However, most of the Middle Palaeolithic assemblages in the region fall with-in the temporal bracket of the Late Pleistocene. In contrast, very meagre evidence from pre-MIS 5 Middle Palaeolithic reflects a huge void in the Indian Palaeolithic record, limiting our understanding of hominin evolution during Middle Pleistocene in the region. The new Middle Palaeolithic site at Retlapalle is very crucial in the context for filling some of the voids. The presence of diverse Levallois products with a few bifacial components show hybrid nature of Retlapalle assemblage, mirroring similar heterogenous assemblages across the subcontinent [26, 32, 38–40]. Future works on Retlapalle will further explore diversity and signs of localization within the broader umbrella of Indian Middle Palaeolithic, with the aim of understanding complex population dynamics during the Middle and Late Pleistocene.

## Supporting information

S1 Table Dose rate data, D_e_ values and OSL ages for the sediment sample Unit E from step trench, Retlapalle. (DOCX)
