# Supplementary material for: Deep-rooted Indian Middle Palaeolithic: Terminal Middle Pleistocene lithic assemblage from Retlapalle, Andhra Pradesh, India
Source: PLoS One. 2024 Aug 27;19(8):e0302580. doi: 10.1371/journal.pone.0302580 (PMC11349113; doi:10.1371/journal.pone.0302580)
Supplement: S1 Table — (DOCX) [file pone.0302580.s001.docx]

Supplementary Information

S1 Table. Dose rate data, D_e_ values and OSL ages for the sediment sample Unit E from step trench, Retlapalle

| Sample Code | Depth (cm) | Radionuclide activity ^a^ | | | | Equivalent doses | | | | OSL  age (ka) |
| --- | --- | --- | --- | --- | --- | --- | --- | --- | --- | --- |
|  |  | U (ppm) | Th (ppm) | K (%) | Total Dose rate^b,c^  (Gy/ka) | No. of aliquots/ grains | Water content (%) | OD (%) | D_e_ (Gy)^d^ |  |
| RTP-18-4 | 265 | 4.08±0.39 | 5.76±1.38 | 0.81±0.09 | 2.64±0.19 | 10 | 12.65 | 30 | 368±39.2 | **139.7±17.9** |

^a^ Radioactivity measurement made on the dried, homogenized and powered sample by gamma-ray spectrometry and alpha counting.

^b^ Includes cosmic-ray dose rate

^c^ 12.5±0.5% and 200±20 ppm Rubidium (^87^Rb) concentrations were used to estimate the internal dose rate

^d^ after subtracting a residual dose of 20 Gy
